# Deep learning approaches for differentiating thyroid nodules with calcification: a two-center study

**DOI:** 10.1186/s12885-023-11456-3

**Published:** 2023-11-23

**Authors:** Chen Chen, Yuanzhen Liu, Jincao Yao, Kai Wang, Maoliang Zhang, Fang Shi, Yuan Tian, Lu Gao, Yajun Ying, Qianmeng Pan, Hui Wang, Jinxin Wu, Xiaoqing Qi, Yifan Wang, Dong Xu

**Affiliations:** 1 Department of Diagnostic Ultrasound Imaging & Interventional Therapy, Zhejiang Cancer Hospital, Hangzhou Institute of Medicine (HIM), Chinese Academy of Sciences, Hangzhou, 310022 China; 2 Wenling Big Data and Artificial Intelligence Institute in Medicine, Taizhou, 317502 China; 3 Taizhou Key Laboratory of Minimally Invasive Interventional Therapy & Artificial Intelligence, Taizhou Campus of Zhejiang Cancer Hospital (Taizhou Cancer Hospital), Taizhou, 317502 China; 4 Zhejiang Provincial Research Center for Cancer Intelligent Diagnosis and Molecular Technology, Hangzhou, 310022 China; 5 Key Laboratory of Head & Neck Cancer Translational Research of Zhejiang Province, Hangzhou, 310022 China; 6 Department of Ultrasound, The Affiliated Dongyang Hospital of Wenzhou Medical University, Dongyang, 317502 China; 7 Capacity Building and Continuing Education Center of National Health Commission, Beijing, 100098 China; 8 Taizhou Campus of Zhejiang Cancer Hospital (Taizhou Cancer Hospital), Taizhou, 317502 China; 9 Department of Ultrasound, Hangzhou Ninth People’s Hospital, Hangzhou, 311225 China

**Keywords:** Deep learning, Thyroid nodule, Ultrasonography, Calcification

## Abstract

**Background:**

Calcification is a common phenomenon in both benign and malignant thyroid nodules. However, the clinical significance of calcification remains unclear. Therefore, we explored a more objective method for distinguishing between benign and malignant thyroid calcified nodules.

**Methods:**

This retrospective study, conducted at two centers, involved a total of 631 thyroid nodules, all of which were pathologically confirmed. Ultrasound image sets were employed for analysis. The primary evaluation index was the area under the receiver-operator characteristic curve (AUROC). We compared the diagnostic performance of deep learning (DL) methods with that of radiologists and determined whether DL could enhance the diagnostic capabilities of radiologists.

**Results:**

The Xception classification model exhibited the highest performance, achieving an AUROC of up to 0.970, followed by the DenseNet169 model, which attained an AUROC of up to 0.959. Notably, both DL models outperformed radiologists (P < 0.05). The success of the Xception model can be attributed to its incorporation of deep separable convolution, which effectively reduces the model’s parameter count. This feature enables the model to capture features more effectively during the feature extraction process, resulting in superior performance, particularly when dealing with limited data.

**Conclusions:**

This study conclusively demonstrated that DL outperformed radiologists in differentiating between benign and malignant calcified thyroid nodules. Additionally, the diagnostic capabilities of radiologists could be enhanced with the aid of DL.

## Background

Thyroid nodules represent a common endocrine disease, with a detection rate of up to 68% in adults [1, 2]. The majority of these nodules are benign and pose minimal risk [3]. However, malignant thyroid nodules can burden patients psychologically, with certain high-risk cases carrying the potential of metastasis. Therefore, it becomes crucial to differentiate between benign and malignant thyroid nodules. Ultrasonography (US) and fine needle aspiration (FNA) constitute the two primary methods for distinguishing between benign and malignant thyroid nodules [4, 5]. FNA, being an invasive procedure, can be significantly burdensome for patients [6]. Hence, US emerges as the preferred evaluation method for thyroid nodule evaluation [3]. During ultrasound examinations, several nodule features, including calcification, a hypoechoic appearance, irregular margins, and a taller-than-wide shape, are strongly correlated with malignancy [2]. Among these features, calcification is present in approximately 19.8–32.1% of all thyroid nodules and is considered one of the most critical ultrasound indicators [7, 8]. However, calcification is not a specific marker for malignancy since it can also occur in benign nodules [9].

Currently, radiologists primarily differentiate between benign and malignant calcified thyroid nodules by assessing the type of calcification present [10]. Calcification can be categorized into three types: microcalcification, macrocalcification, and peripheral calcification [11]. Among these types, microcalcification carries the highest risk of malignancy. The American College of Radiology classifies thyroid nodules with echogenic foci in ultrasound images into three groups: macrocalcification is assigned one point, peripheral calcification is assigned two points, and the presence of punctate echogenic foci is assigned three points [12]. The higher the point value, the greater the associated risk of malignancy [13]. However, both ultrasound imaging and US-FNA methods have limitations in determining the type of calcification. Although microcalcification (indicated by punctate echogenic foci) exhibits the highest correlation with malignancy, nodules with microcalcification are not necessarily malignant [14–16]. Furthermore, nodules exhibiting either macrocalcification or peripheral calcification cannot be automatically classified as benign [17]. In conclusion, the clinical significance of various calcification types remains unclear, underscoring the need for a more objective approach to distinguishing between benign and malignant thyroid nodules featuring calcification.

In recent years, there has been a growing interest in artificial intelligence (AI), particularly in the context of deep learning (DL) and its automatic image analysis capabilities. Liu et al. [18] developed a combined DL model with a high discrimination ability for predicting malignant microcalcification in Breast Imaging-Reporting and Data System (BI-RADS) 4 breast nodules, outperforming junior doctors. Patel et al. [19] utilized DL segmentation and computed tomography radiomics to evaluate the microarchitectural changes in cardiovascular calcification following in vivo interventions. Nam et al. [20] developed DL algorithms capable of detecting calcification in chest radiographs, while Yao et al. [21] developed a multimodal DL model for predicting cervical lymph node metastasis in papillary thyroid carcinoma. However, limited studies currently apply DL techniques to predict the malignancy risk associated with calcified thyroid nodules.

The risk of malignancy linked to various types of calcifications in thyroid nodules remains inconclusive, and the clinical significance of calcification has not been adequately studied. Therefore, our study employs DL techniques to automatically extract malignant features from ultrasound images of calcified thyroid nodules and predict the risk of malignancy. Our objective is to compare the diagnostic performance of our method with that of radiologists and investigate whether it can enhance the diagnostic capabilities of radiologists in making accurate clinical decisions for patients with thyroid nodules.

## Methods

### Study design and datasets

This retrospective diagnostic study was conducted at two centers and included patients who met the following criteria: (1) age 18 years or older; (2) ultrasonographic diagnosis of thyroid nodules; (3) detection of calcification in both ultrasound and pathology reports; and (4) a definitive pathological diagnosis of either benign or malignant nodules (confirmed through surgical specimen or FNA [Bethesda II or VI]). Two pathologists made the pathological diagnoses, and in case of any disagreement, the diagnosis of a third senior pathologist was used. Ultrasound images of low quality with incomplete lesions were excluded after screening.

Written informed consent from the patients was waived by the ethics committee of the Independent Ethics Committee of Zhejiang Cancer Hospital and the Medical Ethics Committee of Taizhou Cancer Hospital (IRB-2020-287, IRB-2023-001-IIT) and all images and data were anonymized.

### Evaluation metrics

The primary outcome measure was to investigate the area under the receiver-operator characteristic curve (AUROC) for diagnosing calcified thyroid nodules. The secondary outcomes included accuracy (ACC), sensitivity (SEN), specificity (SPE), positive predictive value (PPV), and negative predictive value (NPV) for calcified thyroid nodules. We compared the diagnostic performance of the DL models with that of radiologists with varying levels of seniority and investigated whether radiologists could improve their diagnostic accuracy with the aid of the DL model.

### Procedures

**Fig. 1:**
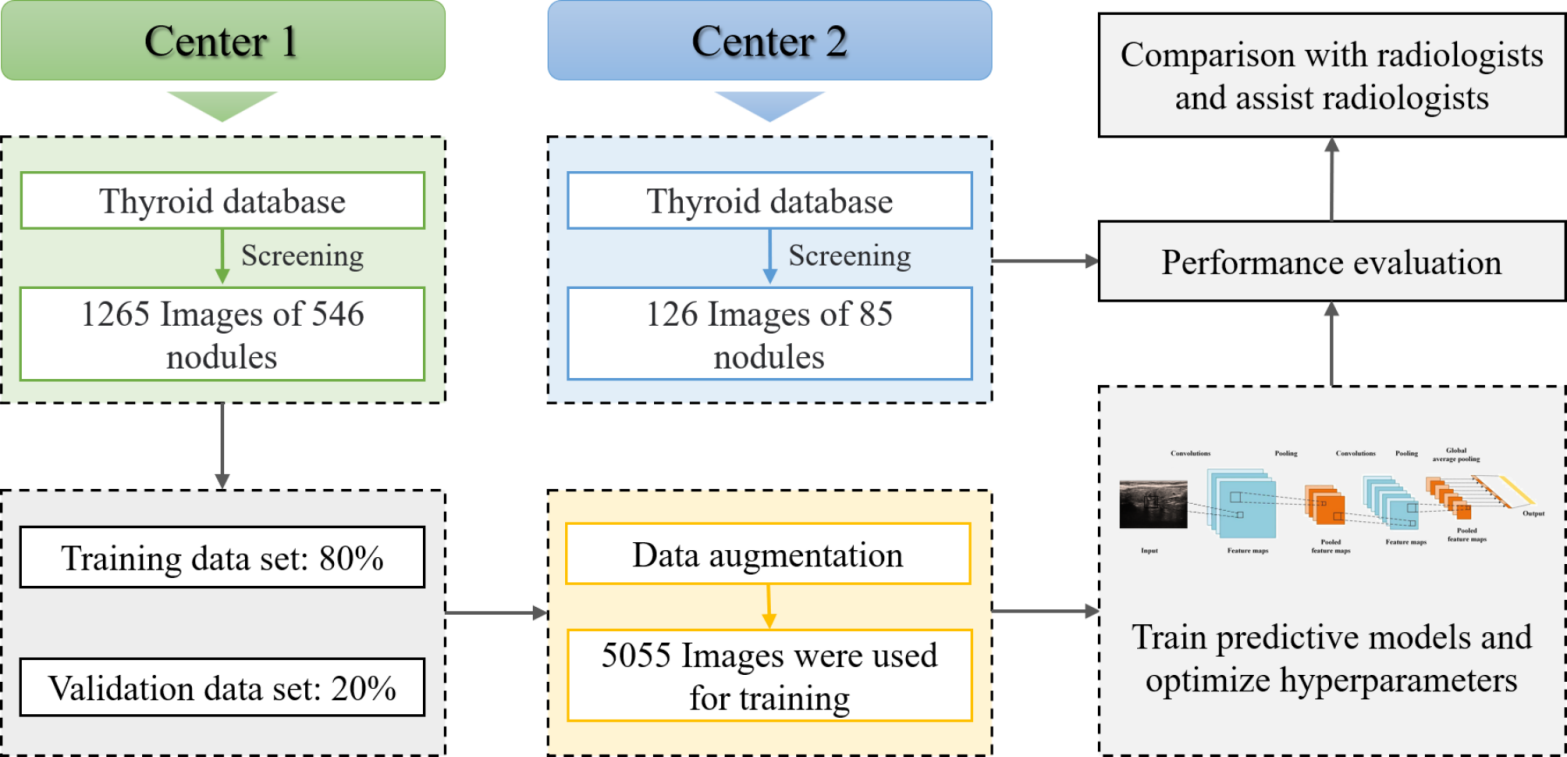
Overall schematic for differentiating between benign and malignant thyroid nodules with calcification using DL models

Figure 1 presents an overall schematic of this retrospective diagnostic study. We conducted a retrospective search in the thyroid ultrasound image databases of both Zhejiang Cancer Hospital (Center 1) and Taizhou Cancer Hospital (Center 2) to identify ultrasound images of patients with thyroid nodules recorded between January 2020 and December 2022. We included images that featured comprehensive ultrasound data and clear pathological results, resulting in a total of 1265 images from 546 nodules from Center 1 and 126 images from 85 nodules from Center 2. We divided the 546 nodules from Center 1 into training and verification sets in an 8:2 ratio. The 85 nodules from Center 2 were designated as a separate test set. Notably, there was no patient overlap between Center 1 and Center 2.

**Fig. 2:**
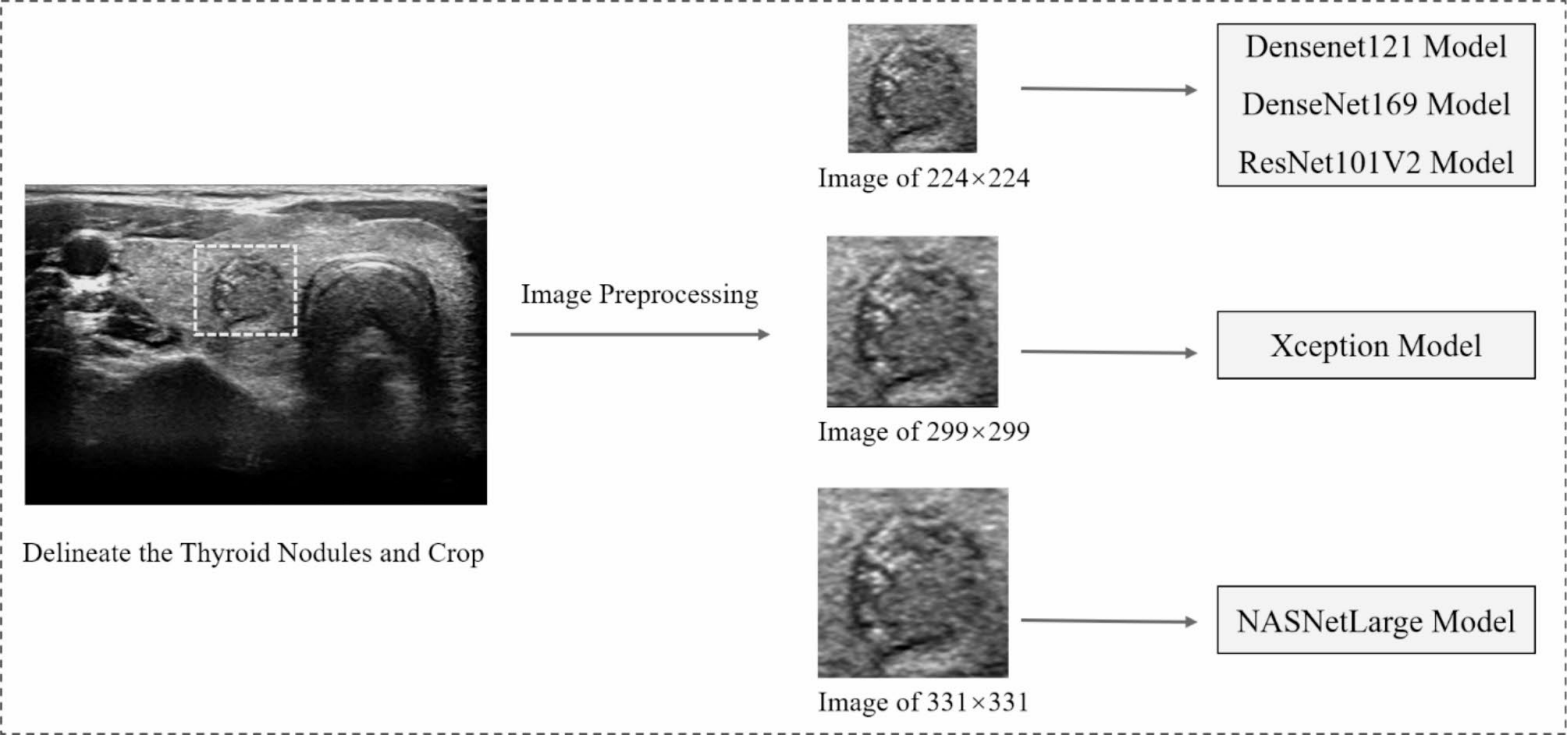
An illustration of the process of inputting images

A total of 1391 ultrasound images of thyroid nodules in Digital Imaging and Communications in Medicine (DICOM) format underwent a lossless conversion to the Joint Picture Group (JPG) format. Manual cropping and removal of noise information, including ultrasound equipment and patient information, from around the original image was performed. As shown in Fig. 2, two radiologists, each possessing over five years of work experience, utilized LabelMe software to outline the nodule area by marking it within a rectangular box on the complete ultrasound image. Following this delineation, a JSON format file containing coordinates, width, and height information for the upper left corner of the nodule was generated. Subsequently, the nodules were extracted from the complete ultrasound image using the JSON format files, resulting in images that only featured the nodule area. To meet the input size requirements of various models, the size of the cropped nodule area image was adjusted accordingly before input into the model. The image length and width were adjusted to 224 × 224, 299 × 299, and 331 × 331 pixels, respectively, to align with the required image input size of the model, and the images were normalized. Due to the limited number of thyroid calcified nodules and the sparse nature of medical data, various data augmentation techniques such as rotation, flipping, scaling, translation, and mixing [22, 23] were applied to transform the existing ultrasound images. These augmentation methods serve to enhance the generalization capabilities of DL models. The ultrasound images of thyroid calcified nodules from Center 1 were divided into a training set (80%) and a validation set (20%). The training set images were expanded to five times their original data volume, which was employed for training the DL models.

In this study, we employed a total of five convolutional neural network (CNN) models, each with distinct structures and depths: DenseNet121, DenseNet169, NASNetLarge, ResNet101v2 and Xception. These models were utilized to extract features from thyroid calcified nodules and establish the classification models. The neural network’s backpropagation method was employed to iteratively update the model parameters, ultimately facilitating the classification of benign and malignant thyroid calcified nodules.

For transfer learning in this study, we utilized the pre-training weight parameters from these models in the ImageNet context. The fully connected layers of the original models’ weight parameters’ were removed and replaced with four fully connected layers containing 1024, 512, 512, and 2 neurons, respectively. During the training process, fine-tuning was applied to expedite the model’s search for the optimal global solution, with the models being iterated for 300 epochs. To prevent overfitting, three dropout regularizations were introduced between the fully connected layers, randomly deactivating 50% of the neurons during training. Binary cross-entropy loss and the Adam optimizer were employed to iteratively update the model’s weight parameters. The initial learning rate for the optimizer was set at 0.001, with a dynamic adjustment strategy. If the ACC did not increase for five consecutive epochs during the training process, the learning rate was reduced, and the learning rate factor was set to 0.5. During training, a batch size of eight was utilized. The SoftMax activation function was applied in the final fully connected classification layer to output the probabilities of benign and malignant thyroid calcified nodules, enabling the DL models to evaluate these nodules. We re-evaluated the generalization performance of the model by conducting 3-fold cross-validation, independently re-adjusting the hyperparameters for each model during this process. Given that we employed DL models with varying convolutional layers and structures, maintaining consistency in hyperparameters across all models was crucial to ensure fair model training. Therefore, all hyperparameters were initialized with default values from the TensorFlow scientific database. The ROC curve was generated from the average probabilities calculated during the three-fold cross-validation, and the remaining metrics were determined through voting to establish the final category results based on the outcomes of the three-fold cross-validation. Two junior and two senior radiologists were tasked with diagnosing 126 identical ultrasound images from Center 2, depicting thyroid nodules, to assess the potential clinical applicability of our model. The four radiologists initially conducted independent diagnoses of the thyroid nodules in the test set. Subsequently, after a washout period exceeding two months, they re-evaluated the same images with the assistance of a DL model. During the initial diagnosis, the radiologists were required to independently identify benign and ma-lignant thyroid calcified nodules. Importantly, the pathological diagnosis for all nodules was established but kept confidential from the radiologists. The radiologists were informed that their diagnostic performance would be compared with that of the DL models. Fol-lowing this initial phase, and after the washout period, we furnished the radiologists with the DL model’s reference diagnosis, which included the malignant probability of nodules and the classification of benign and malignant nodules based on the DL model’s as-sessment. The radiologists had the option to either adhere to their initial diagnosis or incorporate the DL model’s results into their final diagnosis. The diagnostic efficacy of the DL model remained undisclosed to the radiologists.

### Statistical analysis

All statistical analyses were conducted using Python (version 3.8.13), Numpy (version 1.22.3), and Scipy (version 1.8.0). Quantitative data were expressed as mean ± SD. The main evaluation index was the AUROC. A 2 × 2 confusion matrix was generated to calculate ACC, SEN, SPE, PPV, NPV and F1-score (F1) [24, 25]. The receiver operator characteristic (ROC) curve was plotted, depicting the true positive rate (SEN) and the true negative rate (SPE). Subsequently, AUROC values were calculated, and the significance of AUROC differences was assessed using the Delong test, with P < 0.05 considered indicative of a significant difference between the two AUROCs.

## Results

### Patients

The study included a total of 631 thyroid nodules, and Table 1 provides an overview of the characteristics of the patients and nodules examined in the study. In Center 1, the mean age of the patients was 51.5 ± 11.6 years, comprising 380 females and 105 males. Meanwhile, Center 2 recorded a mean patient age of 54.5 ± 11.0 years, encompassing 58 females and 22 males. The average nodule size in Center 1 measured 1.3 ± 1.1 cm, with 72.7% of the nodules being smaller than 1.5 cm. The malignancy rate for nodules in Center 1 was 54.6%. Similarly, Center 2 reported an average nodule size of 1.4 ± 1.2 cm, with 69.4% of the nodules being smaller than 1.5 cm. The malignancy rate for nodules in Center 2 was 58.8%. Table 1 presents an overview of the image distribution within the dataset.

**Table 1:** Patient and Nodule Characteristics*

	Center 1		Center 2
**Age (years)**	51.5 ± 11.6		54.5 ± 11.0
**Sex**			
Female	78.4(380/485)		72.5(58/80)
Male	21.6(105/485)		27.5(22/80)
**Nodule size(cm)**	1.3 ± 1.1		1.4 ± 1.2
< 1.0 cm	53.3(291/546)		45.9(39/85)
1.0 to < 1.5 cm	19.4(106/546)		23.5(20/85)
1.5 to < 2.0 cm	9.0(49/546)		11.8(10/85)
≥ 2.0 cm	18.3(100/546)		18.8(16/85)
**Final pathological**			
benign	45.4(248/546)		41.2(35/85)
malignancy	54.6(298/546)		58.8(50/85)
**Distribution of images** **(Original images/Augmentation images)**	**Training** **data set**	**Validation** **data set**	**Test** **data set**
benign	562/2810	141/-	58/-
malignancy	449/2245	113/-	68/-

*Unless otherwise noted, values represent percentages

### Performance of models

**Fig. 3:**
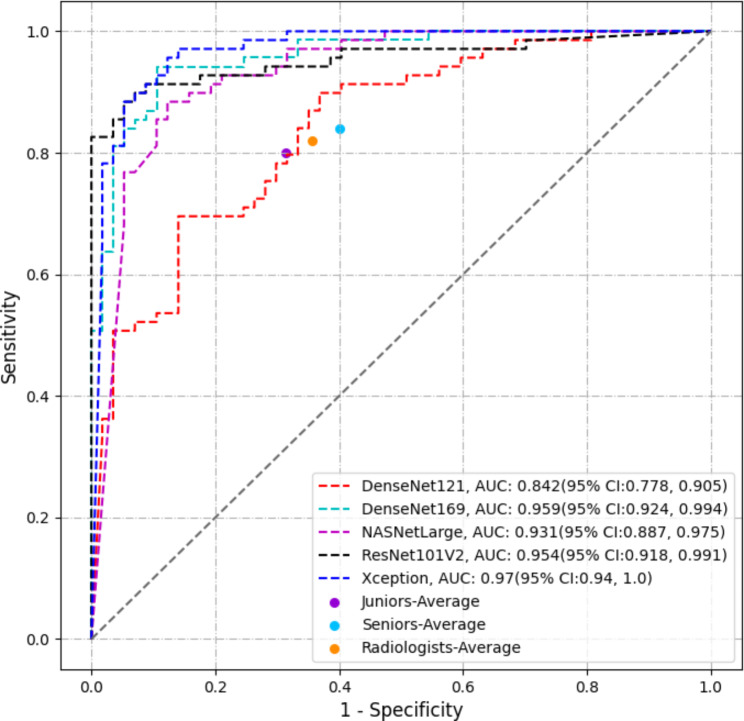
ROC curves of the DL models and the performance of radiologists aided by the Xception model

After 300 epochs of learning, the external test set from Center 2 was employed to evaluate the performance of the five classification models. The Xception classification model exhibited the highest performance, achieving an AUROC of up to 0.970, followed by the DenseNet169 model with an AUROC of up to 0.959. The key distinction between the Xception model and the other four models lies in its incorporation of deep separable convolution into the model structure. This structural element segregates the channel axis of the image data from the spatial axis and subsequently conducts separate convolutions on these two channels. This separation framework results in a reduction in the model’s parameter count, reduces computational complexity, and enhances overall efficiency. Furthermore, this structure enhances the model’s capacity to capture both local features and global information within the image, ultimately elevating the model’s ability to comprehend image content. Given its fewer parameters and stronger feature extraction capabilities, the Xception model exhibits superior generalization performance and overall performance, particularly when dealing with limited data samples. Figure 3 illustrates the AUROC curves for all five models, while Table 2 provides an overview of the evaluation metrics for each model.

**Table 2:** Diagnostic performance of DL models in the test set

	Densenet121	DenseNet169	NASNetLarge	ResNet101V2	Xception
ACC (95% CI)	0.741 (0.648, 0.834)	0.906 (0.844, 0.968)	0.882 (0.814, 0.951)	0.941 (0.891, 0.991)	0.929 (0.875, 0.984)
SEN (95% CI)	0.886 (0.818, 0.953)	0.857 (0.783, 0.932)	0.857 (0.783, 0.932)	1.000 (1.000, 1.000)	0.914 (0.855, 0.974)
SPE (95% CI)	0.640 (0.538, 0.742)	0.940 (0.890, 0.990)	0.900 (0.836, 0.964)	0.900 (0.836, 0.964)	0.940 (0.890, 0.990)
PPV (95%CI)	0.633 (0.530, 0.735)	0.909 (0.848, 0.970)	0.857 (0.783, 0.932)	0.875 (0.805, 0.945)	0.914 (0.855, 0.974)
NPV (95%CI)	0.889 (0.822, 0.956)	0.904 (0.841, 0.967)	0.900 (0.836, 0.964)	1.000 (1.000, 1.000)	0.940 (0.890, 0.990)
AUC (95%CI)	0.842 (0.778, 0.905)	0.959 (0.924, 0.994)	0.931 (0.887, 0.975)	0.954 (0.918, 0.991)	0.970 (0.940, 1.000)
F1	0.744	0.922	0.900	0.947	0.940

### Performance of radiologists with and without DL assistance

The Xception classification model outperformed other models, closely followed by the DenseNet169 model (Table 2). Table 3 presents the performance of both junior and senior radiologists in diagnosing benign and malignant thyroid calcified nodules. The combined AUROC of the two junior radiologists was 0.674, while that of the two senior radiologists was 0.745. These findings indicate that both models outperformed the radiologists, with a statistically significant difference (a P-value < 0.05, as indicated in Table 4).

**Table 3:** Diagnostic performance of four radiologists

	Radiologist 1 (Junior)	Radiologist 2 (Junior)	Juniors Average	Radiologist 3 (Senior)	Radiologist 4 (Senior)	Seniors Average
ACC (95% CI)	0.706 (0.609,0.803)	0.682 (0.583,0.781)	0.694 (0.596, 0.792)	0.765 (0.675,0.855)	0.729 (0.635,0.823)	0.747 (0.655, 0.839)
SEN (95% CI)	0.514 (0.408,0.620)	0.600 (0.496,0.704)	0.557 (0.451, 0.663)	0.800 (0.715,0.885)	0.657 (0.556,0.758)	0.729 (0.635, 0.823)
SPE (95% CI)	0.840 (0.762,0.918)	0.740 (0.647,0.833)	0.790 (0.703, 0.877)	0.740 (0.647,0.833)	0.780 (0.692,0.868)	0.760 (0.669, 0.851)
PPV (95%CI)	0.692 (0.594,0.790)	0.618 (0.515,0.721)	0.655 (0.554, 0.756)	0.683 (0.584,0.782)	0.676 (0.577,0.775)	0.680 (0.581, 0.779)
NPV (95%CI)	0.712 (0.616,0.808)	0.725 (0.630,0.820)	0.719 (0.623, 0.815)	0.841 (0.763,0.919)	0.765 (0.675,0.855)	0.780 (0.692, 0.868)
AUC (95%CI)	0.677 (0.578,0.776)	0.670 (0.570,0.770)	0.674 (0.574, 0.774)	0.770 (0.681,0.859)	0.719 (0.623,0.815)	0.745 (0.652, 0.838)
P-value	All P < 0.05					

**Table 4:** The p-values of the DeLong test for different methods in the test set

	DenseNet169	Xception	Radiologists(Junior)	Radiologists(Senior)
DenseNet169e	1	0.4516	0.0022	0.0192
Xception	0.4515	1	0.0021	0.0019
Radiologists (Junior)	0.0022	0.0021	1	0.2216
Radiologists (Senior)	0.0192	0.0019	0.2216	1

Table 3 and Table 5 provide insight into the diagnostic performance of radiologists in distinguishing between benign and malignant thyroid calcified nodules, both with and without the assistance of the Xception model. In the absence of the DL model, the combined AUROC for the two junior radiologists was 0.674 (0.574, 0.774). However, with the DL model’s assistance, the AUROC for the junior radiologists increased to 0.743 (0.650, 0.836). The combined ACC also improved from 0.694 (0.675,0.855) to 0.753 (0.662, 0.845). Similarly, when not aided by the DL model, the combined AUROC for Radiologist 4 was 0.719 (0.623, 0.815), and with the DL model’s assistance, it increased to 0.764 (0.674, 0.855). The ACC also improved from 0.729 (0.635, 0.823) to 0.788 (0.701, 0.875).

**Table 5:** Diagnostic performance of four radiologists aided by the Xception model

	Radiologist 1 (Junior)	Radiologist 2 (Junior)	Juniors Average	Radiologist 3 (Senior)	Radiologist 4 (Senior)	Seniors Average
ACC (95% CI)	0.729 (0.635, 0.824)	0.776 (0.688, 0.865)	0.753 (0.662, 0.845)	0.694 (0.596, 0.792)	0.788 (0.701, 0.875)	0.741 (0.649, 0.834)
SEN (95% CI)	0.657 (0.556, 0.758)	0.714 (0.618, 0.810)	0.686 (0.587, 0.784)	0.571 (0.466, 0.677)	0.629 (0.526, 0.731)	0.600 (0.496, 0.704)
SPE (95% CI)	0.780 (0.692, 0.868)	0.820 (0.738, 0.902)	0.800 (0.780, 0.885)	0.780 (0.692, 0.868)	0.900 (0.836, 0.964)	0.840 (0.764, 0.916)
PPV (95%CI)	0.676 (0.577, 0.776)	0.735 (0.642, 0.829)	0.706 (0.677, 0.803)	0.645 (0.543, 0.747)	0.815 (0.732, 0.897)	0.730 (0.638, 0.822)
NPV (95%CI)	0.765 (0.675, 0.855)	0.804 (0.720, 0.888)	0.785 (0.765, 0.872)	0.722 (0.627, 0.817)	0.776 (0.687, 0.865)	0.749 (0.657, 0.841)
AUC (95%CI)	0.719 (0.623, 0.814)	0.767 (0.677, 0.857)	0.743 (0.650, 0.836)	0.676 (0.576, 0.775)	0.764 (0.674, 0.855)	0.720 (0.625, 0.815)
P-value	All P < 0.05					

### Attention maps generated by CAM

**Fig. 4:**
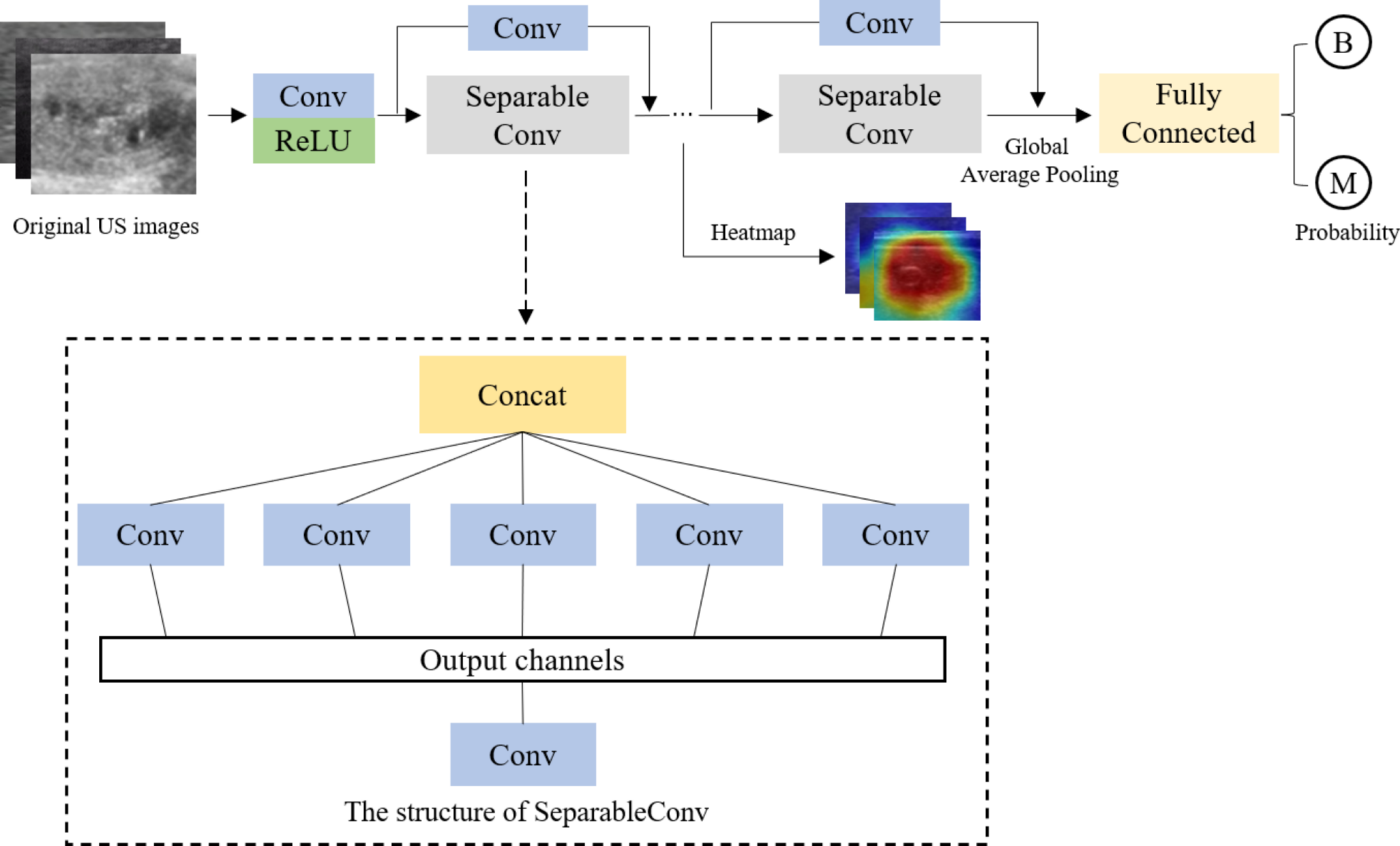
Workflow diagram of the Xception model, “B” stands for benign and “M” for malignant

**Fig. 5:**
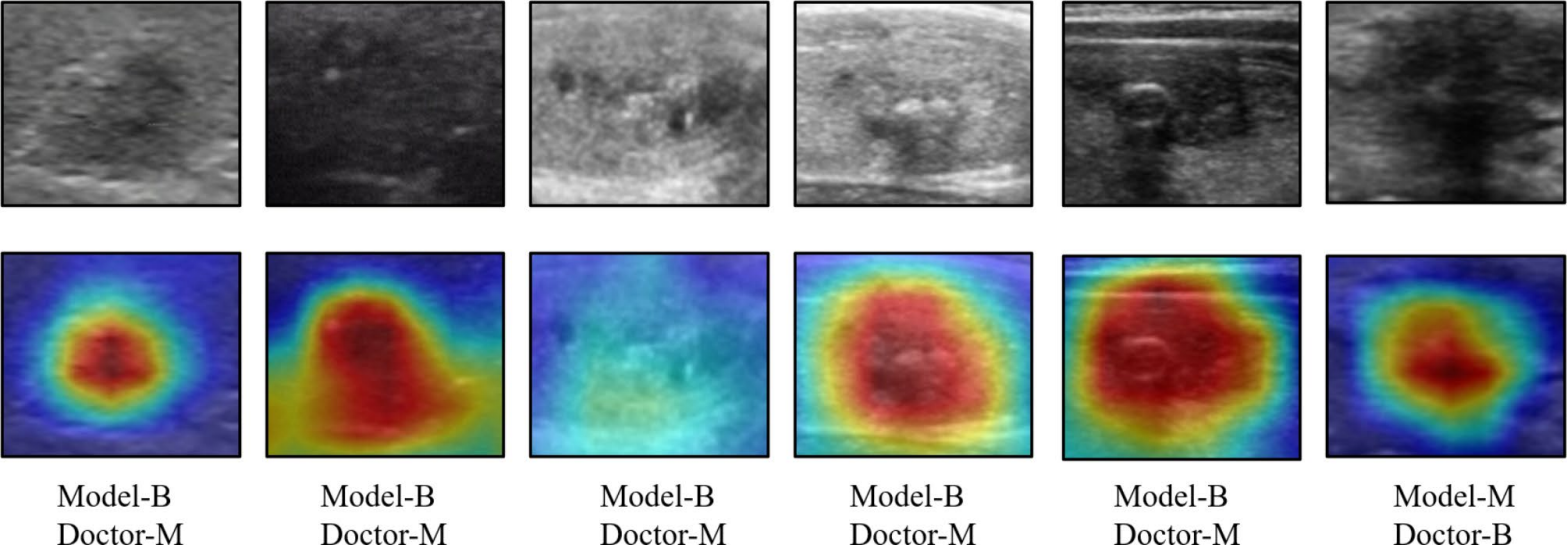
CAM-Generated attention maps, “B” stands for benign and “M” for malignant

Upon forwarding the image of the calcified thyroid nodule to the Xception model, a convolution operation is performed, followed by the application of the Rectified Linear Unit (ReLu) activation function to nonlinearly process the extracted features. Subsequently, the image is subjected to depth-separable convolution for further feature extraction. Layer skip connections are incorporated within this process to augment feature utilization. After multiple iterations of this layer, global average pooling (GAP) is employed to reduce the dimensionality of the features. A fully connected layer operates on the features post-global average pooling, facilitating feature learning and classification prediction, ultimately producing the benign and malignant probability scores for calcified nodules. The Xception model workflow is illustrated in Fig. 4. Throughout this process, we implemented the class activation mapping (CAM) model to generate attention maps highlighting the critical regions in the prediction of nodules. Figure 5 displays these maps, with the red areas indicating the regions of primary focus for the DL model. These selected nodules were instances where doctors’ judgments were incorrect, but the model made accurate assessments.

## Discussion

This study evaluated various methods for differentiating between benign and malignant thyroid calcified nodules. Results from the external test set demonstrated that the Xception classification model achieved the highest performance with an AUROC of up to 0.970, followed by the DenseNet169 model with an AUROC of up to 0.959. The Xception classification model, which demonstrated superior generalization ability, provided valuable assistance to radiologists of varying experience levels in differentiating between benign and malignant calcified nodules. These findings underscore the diagnostic potential of DL methods in assisting radiologists to make more accurate clinical diagnoses, potentially reducing the need for unnecessary FNA procedures for thyroid calcified nodules. Typically, DL models outperform radiologists due to their ability to discern intricate details beyond human perception. In this study, we employed the Gradient-CAM technique to generate attention maps, highlighting regions of interest for the CNN. In the future, it is anticipated that additional feature visualization methods for DL models can be utilized to visualize areas of concern, thus enabling the model to directly communicate to doctors which features it deems significant when rendering judgments.

Calcification serves as a common indicator in thyroid nodules, and distinct types of calcifications suggest varying degrees of malignancy. Previous studies have reported that malignant calcification in thyroid nodules typically arises from the proliferation of blood vessels, dense fibrous tissue, and the accumulation of calcium salts [10]. Calcification can be present in both benign and malignant thyroid nodules, and the morphological features often overlap [26]. In postoperative histopathological examinations, microcalcification is associated with the presence of psammoma bodies, which are round, lamellar, crystalline calcified deposits measuring between 10 and 100 μm in size and are distinctive features unique to papillary thyroid carcinoma. Hence, microcalcifications are highly indicative of malignancy, with an SPE ranging from 86 to 95% and a PPV varying between 42% and 94% [27]. However, the identification of microcalcifications in ultrasound images is highly dependent on the scanning angle and the radiologists’ experience, and outcomes may differ across varying imaging machines. Regarding peripheral calcification, certain studies [4, 28, 29] have suggested that the disruption and thickening of peripheral calcification, along with the presence of a peripheral halo around thyroid nodules, strongly indicate a heightened likelihood of malignancy. However, several other studies [17, 30] refute this conclusion, possibly due to the subjective interpretation of radiologists regarding peripheral calcification [30, 31]. Similarly, previous studies have reported conflicting and inconsistent results regarding the malignant risk associated with macrocalcification [32–37]. This variability may stem from variations in the composition and echogenic characteristics displayed in ultrasound images of thyroid nodule. Discrepancies may also arise from differences in how radiologists define distinct calcification types and the specific attributes of the study populations. Ha et al. [37] integrated the Thyroid Imaging, Reporting and Data System (TI-RADS) to stratify the malignant risk of thyroid nodules with different echogenic foci types. Their findings revealed that the PPV for nodules featuring large echogenic foci without shadowing, macrocalcification, peripheral curvilinear or eggshell echogenic foci, with or without shadowing, was relatively low (33.3–56.4%). However, when the highly suspicious categories within TI-RADS were combined, the PPV notably increased to a range of 50.0–90.9%. In our study, the PPV achieved by the DenseNet169 and Xception models could reach as high as 90.9% and 91.4%, surpassing the traditional combination of TI-RADS for assessing the malignant risk associated with calcified nodules.

Both US and FNA have limitations in determining the malignant risk of thyroid calcified nodules. FNA, for instance, can only observe morphological and structural changes in a limited number of cells. Additional constraints include a limited comprehension of the overall tissue structure and the potential for undetected cases due to unsatisfactory sampling. Consequently, there is a pressing need for supplementary diagnostic methods. DL methods offer potential advantages over traditional diagnostic techniques due to their objectivity and reproducibility. These methods operate by automatically classifying images through the training of CNNs on extensive datasets. A CNN comprises multiple convolutional layers capable of automatically extracting meaningful features from input data and integrating them as they traverse through deep layers. Specifically, CNNs excel at automatically classifying images by extracting optimal features, identifying, and analyzing the characteristics of thyroid nodules, and effectively distinguishing between benign and malignant nodules [38, 39]. Given that some radiologists may overestimate the malignant risk associated with calcification, it becomes crucial to maintain a high SPE in the identification of thyroid calcified nodules to minimize unnecessary FNA or surgery for benign calcified nodules. The DL method proposed in this study demonstrates its ability to significantly enhance the diagnostic accuracy of radiologists with varying levels of experience in distinguishing between benign and malignant thyroid calcified nodules, potentially averting unnecessary invasive procedures for benign calcified nodules. A distinguishing feature of the Xception model compared to the other four models lies in the introduction of deep separable convolution within the model structure. This structural enhancement translates to fewer parameters and reduces computational complexity compared to the other four models. Simultaneously, this structural element enhances the model’s capability to capture features across different scale feature maps during feature extraction, thereby strengthening its capacity to comprehend and convey image content. Furthermore, this enhancement allows the Xception model to exhibit robust learning capabilities even with limited sample data, achieving superior performance with the same volume of data. Although the AUROC of the Xception model reached 0.970, surpassing the performance of the other models and radiologists with varying levels of experience, the radiologists’ performance did not match or exceed that of the Xception model, even when aided by the DL model. Radiologists displayed only marginal improvements in diagnostic proficiency when comparing the results with and without the DL model’s assistance. This divergence could potentially be attributed to radiologists’ lack of awareness regarding the performance of our DL model during their second diagnosis, fostering a heightened sense of confidence and subjectivity. The noticeable decline in performance exhibited by Radiologist 3 further underscores the significant subjectivity that can persist among radiologists during the process of assisted image interpretation. In future studies, we intend to inform radiologists of the model’s results, which may lead to improved DL-assisted image interpretation. Additionally, it is worth noting that we only provided static images, and the DL model could identify numerous features not discernible to the naked eye. Nonetheless, the limited cut surface may have resulted in the loss of valuable information for radiologists. As demonstrated in Tables 3 and 5, junior radiologists were able to match or even surpass the performance of the senior radiologists with the assistance of the DL model.

Our study has several limitations that warrant acknowledgment. Firstly, selection bias was inevitable as we exclusively included calcified nodules with confirmed pathological diagnosis. A significant proportion of benign nodules do not undergo pathological examination, potentially contributing to the higher incidence of malignancy observed in our study. Secondly, the static ultrasound images used in this study offer a limited angle for viewing thyroid nodules, possibly resulting in lower ACC for radiologists compared to dynamic video identification. Future studies should consider including dynamic videos to enhance accuracy. Thirdly, while radiologists were tasked with assessing benign and malignant thyroid nodules in our study, in clinical practice, radiologists may prescribe FNA for certain suspicious benign nodules. Consequently, the performance of radiologists in our study may have been overestimated. Finally, our study specifically focused on thyroid calcified nodules, thus neglecting consideration of other non-calcified nodules. Early nodule screening relies heavily on the expertise of radiologists and the capabilities of imaging equipment, especially given the small size and concealed location of nodules. Our approach, being an AI method based on ultrasound images, is inherently limited in this regard. Alternatively, the utilization of T-cell receptor (TCR) sequencing data offers a promising avenue for early cancer diagnosis and demonstrates greater generalizability than our approach, which solely targets thyroid calcified nodules [40–42]. However, this method is expensive and does not accurately pinpoint the nodule’s location, potentially impacting subsequent treatment planning. In the future, it would be prudent to integrate TCR data with existing imaging screening methods and assess whether it can elevate the diagnostic proficiency of doctors.

## Conclusions

In conclusion, our study trained and validated DL models using 1265 images of 546 nodules in Center 1, with an external test set of 126 images of 85 nodules from Center 2. Our findings affirm that DL methods outperform radiologists in the evaluation of thyroid nodules with calcification, establishing them as valuable adjunctive tools. However, further training and validation on multicenter data are necessary before integrating this method into clinical practice.

## Data Availability

The datasets generated and/or analysed during the current study are not publicly available due privacy but are available from the corresponding author on reasonable request. The neural networks used in our AI system were developed in Tensorflow2.4.0-GPU. Code for preprocessing the data and running the inference, including the weights of the neural networks, sufficient to evaluate our system on other datasets, is available for research purposes upon a request made to the corresponding author (xudong@zjcc.org.cn). Requests will be answered within one week. At this point, we are not sharing the code publicly in order not to compromise potential commercialization of our system.

## List of abbreviations

ACC: Accuracy
AI: Artificial intelligence
AUROC: Area under the receiver-operator characteristic curve
BI-RADS: Breast Imaging-Reporting and Data System
CAM: Class activation mapping
CNN: Convolutional neural network
DICOM: Digital Imaging and Communications in Medicine
DL: Deep learning
F1: F1-score
FNA: Fine needle aspiration
GAP: Global average pooling
JPG: Joint Picture Group
NPV: Negative predictive value
PPV: Positive predictive value
ReLu: Rectified Linear Unit
ROC: Receiver operator characteristic
SEN: Sensitivity
SPE: Specificity
TCR: T cell receptor
TI-RADS: Thyroid Imaging, Reporting and Data System
US: Ultrasonography
